# “It’s something that marks you”: Abortion stigma after decriminalization in Uruguay

**DOI:** 10.1186/s12978-018-0597-1

**Published:** 2018-09-10

**Authors:** Roosbelinda Cárdenas, Ana Labandera, Sarah E. Baum, Fernanda Chiribao, Ivana Leus, Silvia Avondet, Jennifer Friedman

**Affiliations:** 1 International Planned Parenthood/Western Hemisphere Region, New York, USA; 2 Hampshire College, Amherst, USA; 3 Iniciativas Sanitarias, Montevideo, Uruguay; 4 Ibis Reproductive Health, Oakland, USA

**Keywords:** Uruguay, Latin America, Abortion stigma, Decriminalization, Abortion

## Abstract

**Background:**

Abortion stigma is experienced by women seeking abortion services and by abortion providers in a range of legal contexts, including Uruguay, where abortion was decriminalized up to 12 weeks gestation in 2012. This paper analyzes opinions and attitudes of both abortion clients and health professionals approximately two years following decriminalization and assesses how abortion stigma manifests among these individuals and in institutions that provide care.

**Methods:**

In 2014, we conducted twenty in-depth, semi-structured interviews with abortion clients (*n* = 10) and health care professionals (n = 10) in public and private facilities across Uruguay’s health system. Interviews were recorded, transcribed, and then coded for thematic analysis.

**Results:**

We find that both clients and health professionals express widespread satisfaction with the implementation of the new law. However, there exist critical points in the service where stigmatizing ideas and attitudes continue to be reproduced, such as the required five-day waiting period and in interactions with hospital staff who do not support access to the service. We also document the prevalence of stigmatizing ideas around abortion that continue to circulate outside the clinical setting.

**Conclusion:**

Despite the benefits of decriminalization, abortion clients and health professionals still experience abortion stigma.

## Plain English summary

Abortion stigma is experienced by women seeking legal induced abortion services and by abortion providers in a range of legal contexts. This paper analyzes abortion stigma in Uruguay, where abortion was decriminalized up to 12 weeks gestation in 2012. In it, we explore the opinions and attitudes of both abortion clients and health professionals approximately two years after decriminalization in order to assess if and how stigma manifests within institutions that provide abortion services.

Respondents were interviewed after participating in a survey at the clinical setting where they provide or receive reproductive health services. Our qualitative analysis found that both clients and health professionals express widespread satisfaction with the implementation of the new law. However, despite clear improvements in quality of care, there exist critical points in the service where stigmatizing ideas and attitudes continue to be reproduced. Overall, we conclude that despite the benefits of decriminalization, abortion clients and health professionals still experience abortion stigma both inside and outside the clinical setting.

## Background

### Abortion stigma

Stigma has been found to be an obstacle in delivery of some health services due to negative consequences for those who are, or who fear being, stigmatized [1, 2]. Erving Goffman, a key figure in the sociological definition of stigma, conceptualizes it as “an attribute that is deeply discrediting” that “reduces an individual from a whole and usual person to a tainted, discounted one” [3]. Goffman presents three types of stigma: blemishes of character, deformations of the body, and group identity [3]. Link and Phelan, in response to the assertion that stigma was “vaguely defined” and “individually focused,” explain it as a social process in which individuals are marked as different, associated with negative attributes, conceived of as “others,” separated from society, and subject to loss of status and discrimination. This process places them in a framework of economic, political, and social power relations that perpetuate stigma in order to maintain the status quo [4].

Every year over 40 million women in the world have an abortion [5], making it one of the most common and safe medical procedures [6]. Yet it is still loaded with strong social stigma expressed in negative attitudes and secrecy by both women who get abortions and clinicians involved in the process. Thus, abortion stigma is one of the main barriers to women seeking termination of an unwanted pregnancy and a challenge to abortion service providers. This stigma translates into shame and silence for women and into marginalization for providers, and creates or perpetuates myths and misunderstandings about abortion [2, 7]. Stigma manifests differently depending on legal frameworks, religious beliefs, and social and cultural contexts [8].

Kumar, Hessini, and Mitchell define abortion stigma as “a negative attribute ascribed to women who seek to terminate a pregnancy that marks them, internally or externally, as inferior to ideals of womanhood” [9]. They explain that when a woman has an abortion, she transgresses socially-accepted concepts, such as that sexual relations are only for reproductive purposes; that maternity is inherent in the condition of being a woman, and therefore inevitable; and that the role established for women is motherhood and the nurturing of children [9].

At the individual level, stigma can be classified into three main manifestations: 1) perceived stigma, which are ideas about what others may think about abortion and about what could happen if their own experience is made public (rejection by the family or partner, impaired social relationships, loss of friendships, criticism, abuse, and isolation); 2) experienced stigma, which are actual acts of discrimination, harassment, and aggression by others; and 3) internalized stigma, which refers to the materialization of the two previous forms in feelings of guilt, shame, anxiety, or other negative ideas [9, 10].

Shellenberg et al. [10] and Sorhaindo et al. [11] focus on how internalized stigma was experienced by women who had abortions in Mexico and Peru. Feelings of guilt, sadness, and shame were common, as well as widespread silence and secrecy around abortion, especially in small communities. Some of the women interviewed by Shellenberg et al. [10] initially said that their abortions were spontaneous, a strategy to distance themselves from stigma, although they later explained that they had induced abortions. Some women in the study by Sorhaindo et al. [11], said that they had changed their perception about abortion based on their own experience. Yet despite changing their prejudiced views of women who terminate their pregnancies, they were unable to approve of abortion even after having one. The accounts in these studies reflect experiences of isolation among women who choose to not share their experiences or seek support [9, 10].

Health care providers who counsel women and dispense abortion medication, as well as pharmacists and other personnel who work in facilities that provide abortion services are also affected by stigma [10–12]. This stigma often discredits them and excludes them from full participation in their professional community. For example, abortion providers have been called “dirty workers” in the social psychology literature. “Dirty work” refers to professions stigmatized by their associations with contamination that is physical (grime, dirt), social (interaction with stigmatized individuals), or moral (primarily sin) [12]. Studies conducted with abortion providers where abortion is illegal reveal that they frequently feel isolated from the general medical community and that they are afraid to speak openly about their work [8, 12]. Under these conditions, many choose not to get involved in abortion provision, or if they do, they do not speak openly about it in their social and professional circles [13].

Even though research that specifically explores how abortion stigma operates among Latin American women and health professionals is incipient, there is a growing body of work that provides solid bases with which to develop theoretical frameworks that are grounded on empirical evidence [10, 11, 16–19].

Although an increasing number of countries Latin America have achieved partial decriminalization of abortion in recent years, it is still estimated that the region has the highest percentage of unsafe abortions in the world [5, 14]. Furthermore, research shows that in settings where abortion is legal, risks to the lives of women and abortion-related stigma are lower than in those where it is criminalized [14, 15]. Thus, this paper emerges from the broad idea that perceptions and attitudes toward abortion vary depending on legal contexts, and that these tend to be more favorable when a woman’s legal right to terminate her pregnancy is recognized. The study’s overall objective was to uncover and analyze these patients’ and health professionals’ perceptions and attitudes towards abortion. Specifically, to explore if and how stigma continues to operate in decriminalized clinical abortion settings.^1^

### Legal framework

In 2012 (Law 18,987), Uruguay decriminalized medicated and surgical abortion, without specifying grounds but under strict compliance with two requirements: to not exceed twelve weeks of pregnancy and that the woman meet with a multidisciplinary team of three health professionals (gynecologist, psychologist, and social worker), which must ensure that she has all necessary information available to make an informed and responsible decision. The law and its regulations (Decree No. 375/012) state that a woman must attend four visits and during the process, the woman must think over her decision for five days except in cases where the pregnancy represents a risk to the woman’s life, is a result of rape, or when there is fetal malformation. In addition, the IVE^2^ manual published by the Ministry of Health following decriminalization privileges medicated abortion over surgical procedures. For this reason, women who go through a regular abortion process will routinely receive a medicated abortion and will not be asked for their preference of method. All public facilities in Uruguay’s Integrated Health System are required to follow the law and conscientious objection is permitted among physicians as long as the facilities where objectors work inform women on how and where to access services.^3^

In the Uruguayan model of care, teams of health professionals in abortion services are organized in first level clinics and in hospitals, and they include physicians, nurses, midwives, psychologists, social workers, and sonographers. Under the law, abortion clients must follow the following steps: a first appointment where the woman expresses her intent to terminate a pregnancy (Visit 1), a second appointment with the interdisciplinary team where she receives counseling and is informed about the required reflection period (Visit 2), a five-day waiting period, a third appointment where the woman expresses her final decision and the procedure is initiated (Visit 3), and a fourth appointment to confirm whether the abortion has been completed (Visit 4).

## Methods

### Recruitment and data collection

In order to test whether the legal recognition of the right to terminate a pregnancy has an impact on stigma, we collected data from a group of abortion clients and a group of health professionals who provide abortion care in Uruguay in 2014, two years following decriminalization.^4^ In Uruguay, women were recruited at the Sexual and Reproductive Health Service of the Complejo Hospitalario Pereira Rossell, a major public hospital in the capital city, Montevideo.^5^ At the end of the second appointment (Visit 2), following counseling by the interdisciplinary team, women were invited by a nurse to participate in the study. Women received necessary information about the research and, if they were interested in participating, provided their verbal consent and contact information to be contacted later. Interviews were conducted in person or by telephone following the fourth appointment (Visit 4). The interviews analyzed in this paper are a subsample of a larger group of abortion clients who completed a survey for a quantitative analysis. Of a total of 203 abortion clients recruited for the quantitative study, ten were chosen by convenience sampling to be interviewed in person. Semi-structured in-depth interviews (from 30 to 90 min long) were conducted with participants aged 22 to 38 years. Although most lived in Montevideo, some respondents lived outside Uruguay’s capital city. Health professionals were also invited to participate in an interview following participation in a quantitative study. Among a sample of 72 health professionals in facilities around the country who completed the quantitative survey, ten completed an in-depth interview including seven women and three men. These included physicians, midwives, social workers, and a psychiatrist. Interviews lasted from 20 to 60 min. Most interviews were conducted in person by trained study coordinators; some were conducted by telephone due to long travel distances. This study was approved by the Allendale Investigational Review Board (Old Lyme, CT, USA).

All interviews were recorded, transcribed, and coded for analysis using Dedoose software. The research team jointly developed two codebooks, one for interviews with health professionals and one for interviews with women. The codebooks were created independently to capture unique topics in the semi-structured interview guides and in vivo codes that emerged directly from the participants; however the codebooks reflected similar overarching themes such as attitudes towards the abortion law, disclosure and interactions with people in their community, and experiences in different phases of the abortion process, among others. Analysis was led by a team of two researchers and two coders who jointly developed a codebook and carried out thematic analysis of the data. Each of the codes was individually scrutinized first and subsequently the team identified key themes and patterns in responses.

## Results

### Opinions and attitudes among abortion clients

#### Attitudes towards abortion and women who have abortions

While some women believe that Uruguay continues to be a conservative country where abortion is still taboo, others said they saw major progress in social attitudes, including the fact that decriminalization had been achieved. Almost all interviewees felt that abortion is their right and that they have the prerogative to decide what to do with their bodies and their lives. In general, abortion is seen as an individual experience that is only incumbent on the person who has to get one. Although most spoke with their partner, family members, or friends, a few did not share the experience with anyone other than the legal abortion team that provided care during the process.

“I have nothing to say; what needed to be said got said and that’s that. I did it, I’m fine, and ciao.” (Age 23).

“I didn’t talk about it with my husband, or my sister, or my friends, or with anybody. The topic is now closed, that’s that.” (Age 34).

Religious ideas were almost nonexistent in interviewees’ discourse. However, they all reported some level of guilt, describing “pangs of conscience,” and other feelings, such as “a non-physical pain,” shame, sadness, anger, depression, and loneliness, and some referred to themselves as selfish. All interviewees shared the idea that the abortion was an experience that marked them for life:

“[…] I think I’m going to carry this until I die.” (Age 23).

“I think it is a trauma that one does not get over, even though one learns to live with it […] this guilt is going to be lifelong.” (Age 22).

“When I think about going to the gynecologist and they are going to ask me about my medical history, I’m ashamed to have to tell them that I just had an abortion.” (Age 38).

Still, one abortion client expressed positive feelings about having access to a legal abortion as compared to an unsafe option:

“It was strange because it was a new experience, but it was a lifesaver. You avoid the trauma of doing it in a clandestine place where they can do anything to you and where no one is going to take responsibility for anything.” (Age 29).

Despite recurring feelings of guilt, none of the participants said they regretted their abortion. However, these clients experienced their process as something that they do not want to have to go through again. In fact, all of them said that, until discovering an unwanted pregnancy, they saw abortion as a very remote possibility, something that they would not have to go through or that they would not be capable of doing.

“[…] I had sworn to myself that I would never have an abortion and I ended up doing it. It was kind of ironic and I was surprised to find myself contradicting myself about something that I said I wouldn’t do.” (Age 23).

“[…] I said it would never happen to me and it happened.” (Age 34).

Several of them said they were against abortion even after having terminated a pregnancy, while others expressed a change of opinion prompted by the experience.

“I’m against abortion, so it was a very difficult decision to make and to this day it still weighs on me.” (Age 31).

“[..] I was against abortion until it was my turn.” (Age 22).

“In line with my upbringing, I had always been against it; I imagined it to be pretty cruel because of what they say and they tell you.” (Age 27).

They all confirmed that it was the best decision they could make, given that it was not the right time to have a child because of various reasons: poor economic or health situations, instability with a partner, or because they already had several children.

Interestingly, with the exception of one interviewee, participants harshly judged other women who have abortions, and even more severely those who have more than one. Although they fully justified their own abortion saying things like “I didn’t want to do it, but of course my situation wasn’t easy” (Age 27), they saw other women’s abortions as acts of irresponsibility, selfishness, and immaturity.

“If you already did it once, why are you going to do the same thing again? Once is fine, but those who do it two, three, four times, [it’s] like they kind of do it just for fun.” (Age 22).

“I see many women who do it like just for fun […] people who say: ‘well, I got pregnant and I can do it one, two, and even three times.’” (Age 31).

“[…] others get pregnant real easy and have abortions [as easily/often as] they change their jeans.” (Age 23).

“[…] there are other cases of women where it’s like they don’t care if they get pregnant and abort and go back and repeat the story […] it’s different for those who want to come every year to do it, but for women whose case is like mine, it’s okay.” (Age 38).

“I think there should be limits because they’re going to get pregnant ten times and they’re going to take the life of an innocent being […] Names and record numbers should be recorded and tell them ‘look, there’s a limit, you can’t get pregnant five times and get rid of it every time you feel like it.’” (Age 22).

#### Attitudes towards abortion health professionals and services

For the women interviewed, the most common feelings before visiting facilities to request an IVE or legal abortion service were shame, fear of rejection, or fear of undergoing a procedure that could endanger their health.

“At first, I was a little afraid of the whole procedure. After the counseling, I felt safe and calm. I felt confident.” (Age 29).

Overall, women had positive experiences with the health care teams at the facility where they received their service. Almost all said that they did not feel judged by the interdisciplinary team. Rather, they said they were surprised by the thorough information they received and that they felt welcomed and well-understood.

“I thought they would be a little more hostile, because of the fact that one is killing a person, but no, everything [was] just fine.” (Age 22).

“You arrive afraid that you’re going to be judged by the same doctors whose job is to save lives, but it was nothing like that, everything super good, marvelous, in fact, they protect you.” (Age 29).

Only one of the clients reported having felt rejected by one of the health professionals who treated her:

“When I came in for the ultrasound, the person who was doing it looked at me and said ‘I don’t do IVE and they looked me up and down. When I gave them the paper, they looked at it and said that the doctor didn’t do IVE and the other went and told them: ‘it doesn’t matter, she’s a patient and you have to take care of her’. She went and took her the paper and the woman came and told me to my face, ‘I don’t do IVE. There were a lot of people outside the examining room and even though not everyone knows what IVE is, it was like […] they were judging me.” (Age 38).

During the interview, women were asked about a hypothetical doctor refusing to provide abortion services. Even though they talked about how disappointing and upsetting it would be for a doctor to disapprove of their decision and said they might have changed their minds had they encountered something like this, they did agree that health professionals should be allowed to object to providing abortion services.

All abortion clients in our sample had a medicated abortion. Despite having expressed a high level of satisfaction with the overall service, many of the women said they would have liked to have been able to choose between a medicated and surgical procedure. Two women said they would have preferred a surgical abortion because they perceived the hospital to be a safer place to have the procedure.

“I thought they were going to do the procedure here, not that it had to be in my home. I was very scared. If I had been given a choice, I would have liked to be admitted because I would have felt calmer” (Age 22).

“When they sent me home to take the pill I thought it was risky and that it would be much better to be seen in a hospital” (Age 22).

#### Opinions on the Uruguay abortion law

Almost all the women interviewed knew about decriminalization of abortion from seeing it on television or in the press, which suggests that the issue had been present in the public discourse. Most found the facility where they obtained abortion services by searching online. All said they were relieved that they did not have to have an illegal or clandestine abortion and saw decriminalization as a major advance with regard to the rights of women in Uruguay. Further, they believed that decriminalization had contributed to changing Uruguayans’ overall attitude towards abortion.

“Since there’s the law, more [people] are in favor [of women’s right to choose].” (Age 22).

“Hopefully no one stops this law, it saves us all, it’s a choice so that one can live as one wants, as one likes. It makes no sense to have children to keep them deprived. It’s a choice they give us to live well.” (Age 29).

These abortion clients became aware of the details of the law and the service—such as the gestational age limit and the abortion method specified in the medical guides—during their first visit to the health care center. When asked about their opinion about the specific aspects of the law, almost all felt that the twelve-week limit was appropriate. Some even felt that it should be earlier due to preconceived—and sometimes inaccurate—views that they held regarding the risks involved and the state of development of the fetus.

“Up to 12 weeks is okay and that is already a lot, because the baby [sic] is already formed and it’s somewhat traumatic, it gives you quite a shock, it had everything: its fingers were formed, even the features on its face. I say that eight weeks would be okay.” (Age 29).

However, the majority of those interviewed were in disagreement with the required five-day reflection period, calling it excessive, unnecessary, and torturous.

“Those five days were endless for me, because when you are sure and you want to be done with it, you want it to be now.” (Age 38).

“If it is already a difficult decision and they make you wait, it becomes torture.” (Age 22).

“You want to be done with it. When they give you that requirement, they don’t strictly consider it to be five days. In my case, it was fifteen days.” (Age 29).

For these women, there was nothing to think over since they had made up their minds before setting foot in the facility for the first time.

### Opinions and attitudes among health professionals who participate in abortion services

#### Attitudes towards abortion and women who have abortions

In general, health professionals who participate in abortion services in Uruguay had positive opinions about the right to choose and they supported women and their choices. Although each health professional has their own limits with regard to the different aspects of the process, all felt that their perspectives should not influence the care they give their patients. The health professionals interviewed said that they saw abortion as a woman’s right and that the decision to terminate a pregnancy is her own business. They believe that each woman has her reasons, that these reasons are worthy, and that their role as medical professionals is not to judge.

“The reasons are worthy; no one can decide what a woman should do or what is best for her.” (Physician).

“Every woman has the right to terminate a pregnancy for whatever reason. I don’t think that it’s right or wrong.” (Physician).

“One doesn’t stop being a person, but my feelings don’t matter when I’m supporting the women. If a woman comes in who is using contraceptives, lives alone, has four children, does not have a pension, and gets pregnant, of course we’ll feel more empathy than for a woman who does not take care of herself, who doesn’t care whether she gets pregnant; but regardless of my feelings and opinions, the decision is the woman’s.” (Physician).

All participants felt that their professional duty was strictly to provide information and services. One health professional expressed this recurrent feeling eloquently by explaining that their professional duty is defined in terms of how they can support the patient’s needs:

“What matters most to me is to support the patient; that’s why I became a gynecologist.” (Physician).

In general, health professionals saw abortions as difficult situations for women; as an experience that no woman wants to have to begin with, and certainly as one that none would want to repeat. Thus, they see abortion overall as a watershed experience in their lives.

“The experience is an abortion in general, whether medicated or surgical. The experience marks a before and an after.” (Physician).

In terms of their opinion of abortion itself, several health professionals saw it simply as one more sexual and reproductive health service; as a routine procedure in their daily professional life, the same as a gynecological exam or a pelvic exam on a pregnant woman. With regard to repeat abortions, most saw them as a result of errors of a medical system that is unable to provide effective training on contraception and the Uruguayan mentality that still sees sexuality as a taboo. But nonetheless, they did not believe that women use abortion as contraception.

“It’s mostly the system’s fault, due to a lack of understanding, time, or information. It is not an ideal situation; it’s not that I like it, but I don’t place the blame on the patient.” (Physician).

We did not find a clear trend regarding health professionals’ preference about whether or not to talk about providing abortion services outside the workplace. Some health professionals talk about it openly:

“I talk about my job with everyone. Family, friends, colleagues. At the family level, sometimes there are differences on the subject of principles or religious values.” (Physician).

While others prefer discretion, noting that they only “talk about [their] job with few people.” (Midwife).

In general, these health professionals expressed great satisfaction with the increase of legal abortion services in the country, without denying that abortion stigma still exists in Uruguayan society. When asked directly about its existence, the interviewed health professionals described this stigma in the following ways:

“[…] it shows in abortion clients’ fear, and is manifested quietly, with rejection and with indifference.” (Physician).

“[…] it exists. I don’t know if it’s to such an extent as to produce consequences. When the law was being defended, a segment of society demonstrated their opposition.” (Physician).

“Many patients who do terminate [pregnancies] want to keep it a secret for fear of what others might say. Where I work, it’s a small town; people gossip about this and are judgmental. These comments affect patients emotionally.” (Physician).

“Uruguay is a stigmatizing society around abortion and a number of other issues.” (Psychiatrist).

#### Attitudes towards health professionals who participate in abortion services

In general, health professionals said they were comfortable with their work teams and believe that their experiences since decriminalization have been positive. Only one health professional reported difficulties in the social context where they work, which is not in the city of Montevideo:

“Since it’s a small town, people ask questions and try to judge, but we try to maintain confidentiality.” (Physician).

One of the most common complaints was that there is great demand for abortion services and few service health professionals:

“[…] the greatest obstacle is patient demand where sometimes we feel a little overloaded; more human resources are needed and more time given to visits.” (Physician).

High demand sometimes hinders the process:

“Delays in the process make [some] women still prefer clandestine abortions. The process is very long; so they prefer to do it outside.” (Midwife).

Although all of those interviewed respected the right of other health professionals to object, they see objection as a significant obstacle, since it can affect women’s decisions and hinder overall service. Some health professionals also mentioned health centers that have not been able to put together abortion teams because in some cases there is a scarcity of gynecologists who are willing to prescribe the abortion medication.

Health professionals felt that administrative staff within hospitals also needed further training, given that, together with the objectors, when they manifest stigmatizing views they too constitute a substantial barrier that negatively impacts the quality of abortion services.

“Improving access to the different services and the interaction among them, for example, with the administrative side, nursing, medical staff, laboratory, they should have periodic meetings to fine-tune issues. Administrators and doctors see voluntary termination of pregnancy in totally different ways.” (Physician).

“Not a single administrator works with us [the IVE teams]; there is a lot of turnover in receptionists and they aren’t trained.” (Midwife).

“Very good work is done on logistics, on timing, coordination. The only detail is with the sonographers, because this needs special handling and technicians are not always trained and can intimidate patients.” (Physician).

The majority of the health professionals who participate in legal abortion services reported having received training before the law went into effect. However, all participants expressed interest in receiving further training in abortion service delivery, including some who want to be trained in vacuum aspiration procedures or care for complications from incomplete abortions.

Health professionals mentioned the bias that exists in the type of abortion procedure they provide, which is almost always medicated abortion. They all confirmed that women are not given a choice between a surgical and medicated procedure. One reason they preferred to provide medicated abortion was because they perceived surgical abortion to have more complications and to cost more.

“It is an option that suits all parties: the woman, because she can do it at home in a setting that is not unfamiliar and if we talk about it as a law, that abortion is medicated and not surgical, it serves the state itself and the different institutions, and health professionals. It’s more economical than hospitalizing all those women.” (Physician).

Others explain the preference for medicated abortion in terms of training, in other words, many health professionals have not been trained to provide aspiration abortions. But in general, most are quite pleased with the widespread use of medicated abortion. Some health professionals also described preference for providing the pills because they could avoid being present during expulsion. Some even said that if they had to perform aspiration abortions they would become objectors.

“If the woman requests the surgical method, I withdraw. I wouldn’t have my colleagues’ support. The situation would get messy. I believe that the woman has to accept that she is the one who is terminating the pregnancy.” (Physician).

“I always say what I do, and that I don’t have to actively participate in the termination. If I had to participate actively I would conscientiously object. I had a very bad experience with a fetus when I was a resident, and I said that I wouldn’t do that again because it was very traumatic.” (Physician).

However, several health professionals believe that patients should be able to choose their preferred abortion method and that health professionals should adhere to their role of providing complete and accurate information on the available options. For most, their primary concern was to not endanger the woman’s life and to prevent complications in order to minimize the risk of empowering their opponents:

“We take care of the woman and we take care of the law because if a complication occurs the opponents will take advantage of it.” (Physician).

#### Opinions on the Uruguay abortion law

Some health professionals believed that following decriminalization in 2012, attitudes in the medical community changed and that the right to choose is increasingly being seen in a better light.

“Yes, I have seen colleagues who were not very convinced that this is a right […], and there are others who are conscientious objectors. But in general, the attitude [towards abortion] is more open now.” (Physician).

“Discussion about the issue is a little more fluid and open; even though you may or may not agree, the woman’s decision is respected.” (Physician).

“Colleagues who were very negative seem calmer now.” (Physician).

Almost everyone believes that implementation of legal abortion services has been very successful across the country. However, the health professionals interviewed outside the capital thought that this success is more obvious in Montevideo and that women outside the capital continued to experience much more difficulty in accessing services.

Opinion was divided on the five-day reflection period. Some think that rather than being a time for reflection, the five days are disrespectful to the patient and stigmatize her, because they challenge a decision that has already been made.

“I don’t think it’s advisable, I think it’s more a contradiction: we say that we respect the patient and then we tell her to go think about her decision.” (Physician).

“Most women have already thought about it and they come in to terminate; very few decide to continue with the pregnancy after counseling.” (Physician).

“There shouldn’t be a set time period. For some it simply does more harm. The person already thought about it before coming to the clinic.” (Midwife).

Some health professionals would prefer to have this requirement removed. Other health professionals believe that it is an adequate period, as long as the wait does not result in exceeding the legal gestational age limit. Others believed that the five days were critical because they enabled women to think without pressure and, in some cases, to continue their pregnancies.

There was consensus that the gestational age limit was appropriate although some believe the limit should be lowered to ten weeks. This is due to the overall view that “[t]he more weeks that go by, the greater the risk to the patient.” (Physician).

## Discussion

Qualitative studies in Uruguay before decriminalization of abortion discussed how the restrictive context was creating high levels of fear, uncertainty, and anxiety among women seeking to terminate a pregnancy. Not only was there a high risk to the health and lives of these women, but some were also exposed to painful emotional experiences and to stigma related to clandestine abortion [20, 21]. This paper sought to take the pulse of this abortion stigma after the legal context changed in Uruguay, following the decriminalization of abortion up to 12 weeks of pregnancy. Therefore, attitudes and perceptions were assessed not only from legal abortion clients, but also from health professionals who participated in abortion services.

It is likely that Uruguay is a unique country in the region in two ways: first, there is a deep commitment by the health professionals who have been advancing the sexual and reproductive rights agenda, in particular through the development and implementation of an innovative harm-reduction model, which helped increase access to safe abortion and usher in decriminalization. Second, in Uruguay there seems to be surprisingly little influence of religious sectors and of religious beliefs on sexual and reproductive health issues. For both abortion clients and health professionals, decriminalization in Uruguay followed logically from this unique history of experimentation with a harm-reduction model that significantly reduced maternal morbidity and mortality. Both health professionals and abortion clients in this study saw decriminalization as a key factor that contributed to mitigating negative social views surrounding abortion and to a substantial reduction of clandestine abortions. In this sense, it is undeniable that decriminalization has contributed towards reducing abortion stigma for both women and health professionals [22, 23].

That said, abortion stigma continues to exist for both women seeking an abortion and health professionals involved in abortion services. In particular, there are several key moments within the service where stigma is more likely to emerge. The first of these involves the mandated five-day reflection period, which almost all clients and some of the health professionals saw as onerous and unnecessary. This requirement has the potential to reproduce stigma because it carries within it an implicit distrust of women’s decision and an assumption that women have not fully considered its consequences before arriving to seek care.

Secondly, the exclusive use of medicated abortion also has the potential to reproduce stigmatizing ideas about abortion as “dirty work.” On the one hand, some health professionals see the preferential use of medicated abortion as a welcome means to avoid hands-on participation in a medicated procedure deemed undesirable. On the other hand, as evidenced by our data, the near-exclusive use of medicated abortion allows some providers to shift the moral and de facto onus of interrupting a pregnancy entirely to women. The implicit message in both of these cases is clear: these professionals prefer to separate themselves from “participating” in abortions, thereby emphasizing the idea that abortion is “dirty work.” Finally, although there is strong scientific evidence to support the prevalent use of medicated abortions [3, 24, 25], the failure to provide women with a choice may negatively impact their experience with the service. In an ideal setting, as our informants corroborate, women would be given an opportunity to choose their preferred abortion method. Since the law permits both types of procedures, it is clear that in the Uruguayan case there is space for additional training that expands medical expertise in the epidemiology and technical aspects of various methods, in particular vacuum aspiration.

Finally, with respect to the operation of stigma within the clinical setting, both health professionals and clients identified clinical personnel who do not participate in abortion services as potential perpetuators of stigma. Clinical personnel who were not involved in abortion services can be divided into two types: broad administrative staff and objectors. The latter were identified as enhancers of stigma when their behavior chastised or otherwise “exposed” clients as wrongdoers. In the case of administrative personnel more broadly, both clients and health professionals identified some of them as de facto gatekeepers who actively obstruct access to abortion services or treat abortion clients differently than clients seeking other services. Health professionals in particular saw this as a shortcoming in the structure of service provision resulting from a lack of training and awareness-raising for hospitals’ technical and administrative personnel with whom women are obliged to interact.

Health professionals were generally comfortable about their jobs in abortion teams and expressed overwhelmingly positive attitudes towards the law. Women also expressed that they felt supported by health professionals who through their compassionate and efficient care often dissipated many of the fears and preconceptions that women held before arriving at the clinic. Health care providers and women benefited from strong professional abortion teams, which provided emotional and technical support to all involved. This aspect of clinical care is reflected in other decriminalized contexts and is a component of the Uruguayan model that should be considered for replication. Both clients and health professionals felt that social attitudes were visibly changing as a result of decriminalization. Future research should continue to document the unfolding of long-term impact of decriminalization on women’s access to safe abortion provision as well as perceived and experienced stigma.

It is clear from our interviews, however, that both women and health professionals believed that abortion is always and necessarily a traumatic experience for women; an experience that marks them for life. That is, although perceptions of the law, of safe abortion, and of the rights of women were generally positive, women and providers still looked down on women that had one or more abortions, saw abortion as a weight that women were to carry for the rest of their lives, and that necessarily marked a before and after. In many of these cases the persistence of guilt or judgment reveals the persistence of social stigma despite the legal change. Thus, it is recommended that interventions designed to reduce stigma should not be limited to clinical settings, but rather should include community-level interventions aimed at interrupting stigmatizing social views, and providing spaces for the reproduction of ideas and practices that normalize abortion and contribute towards further de-stigmatization.

Finally, we would like to point to some of the limitations of this study. In particular, the fact that women were interviewed immediately following their fourth visit meant that the data cannot track whether attitudes towards abortion changed substantially over longer periods of time. It is also important to note that we only interviewed women and providers involved in legal abortion services and therefore this study cannot speak towards the circulation of stigma in clandestine settings.

## Conclusion

Overall, this study reveals that although decriminalization does contribute to mitigating abortion stigma, even in legal clinical settings, there can remain practices that perpetuate the notion that abortion is a dirty and morally questionable practice. For example, the refusal to carry out surgical procedure replicates the idea that direct involvement is “dirty work.” Medicated abortion devolves the moral responsibility to women and since it is the only method available de facto, it limits opportunities for women to choose the way the termination will be carried out. Likewise, the five-day reflection period questions a woman’s motives and would seem to implicitly suggest that the ideal decision would be to continue the pregnancy. Further, while abortion clients reported high levels of satisfaction with services, we have identified several key points where stigma is prone to reappearing in service delivery. For example, stigma affects services when women interact with personnel that do not participate in the abortion teams and who are not sensitive to the issue, or when women are unable to obtain care when encountering objectors. Among women and health professionals alike the idea prevails that abortion is something that leaves a mark, and in the case of women this is accompanied by a negative moral judgment toward other women who have abortions, in particular those who have more than one abortion, and those who do not show remorse. Yet, almost all women said that they themselves did not feel judged during the procedure and the health professionals stated that they did not feel rejected by their colleagues, including those are objectors. It is important to underscore that the legal framework has had a decisive impact on abortion stigma and greatly improves women’s experience seeking abortion care.

## Footnotes

1 Hereon after, we will refer to abortion providers as health professionals who participate in abortion services, or simply as health professionals. We do this in order to highlight the broad spectrum of work that these professionals are involved in, rather than pigeonholing their work as simply abortion provision.

2 IVE is the acronym for *Interrupción Voluntaria del Embarazo* (Voluntary Interruption of Pregnancy), which has become a term in its own right in Uruguay, and is often used instead of the word abortion. Accordingly, IVE1, IVE 2, IVE3 and IVE 4 are used to refer to each of the four mandatory clinical visits established by the clinical guides. In this piece, we primarily use the word abortion but have opted to keep IVE when its meaning is not fully captured by the term abortion. In addition, we have used Visit 1, 2, 3, and 4 to substitute for the IVE terminology described above.

3 Note that in August 2015, a ruling by the Court of Administrative Disputes overturned 7 of the 11 articles in the decree, which were challenged by a group of gynecologists arguing that it restricted their right to conscientious objection. As a result of this ruling, the scope of objection was expanded to include not only gynecologists who prescribe the drug, but also other health providers (e.g., sonographers and nurses) who are involved in preparatory procedures and consultations prior to the termination where information and counseling are provided (Visits 1 and 2).

4 It bears to note that in Uruguay abortion outside the health system continues to be penalized.

5 Within the hospital, recruitment and analysis was done in partnership with *Iniciativas Sanitarias*, a leading coalition of sexual and reproductive health professionals whose work advances sexual and reproductive health and rights as a basic human right for women and men in Uruguay.
